# Is There a Link between Vomeronasalitis and Aggression in Stable Social Groups of Female Pigs?

**DOI:** 10.3390/ani12030303

**Published:** 2022-01-26

**Authors:** Pietro Asproni, Eva Mainau, Alessandro Cozzi, Ricard Carreras, Cécile Bienboire-Frosini, Eva Teruel, Patrick Pageat

**Affiliations:** 1 Department of Tissue Biology and Chemical Communication, Research Institute in Semiochemistry and Applied Ethology (IRSEA), 84400 Apt, France; 2 Department of Animal and Food Science, School of Veterinary Science, Universitat Autònoma de Barcelona, 08193 Barcelona, Spain; eva.mainau@uab.cat; 3 Research and Education Board, IRSEA, 84400 Apt, France; a.cozzi@group-irsea.com (A.C.); p.pageat@group-irsea.com (P.P.); 4 Institute of Food and Agriculture Research and Technology (IRTA), Veïnat de Sies, Monells, 17121 Girona, Spain; ricard.carreras@uvic.cat; 5 Department of Molecular Biology and Chemical Communication, IRSEA, 84400 Apt, France; c.frosini@group-irsea.com; 6 Statistical Analysis Service, IRSEA, 84400 Apt, France; e.teruel@group-irsea.com

**Keywords:** behavior, chemical communication, inflammation, pathology, pig, vomeronasal organ

## Abstract

**Simple Summary:**

The vomeronasal organ plays a key role in intraspecific animal behavior since it is able to detect pheromones. Induced and spontaneous lesions of this structure have been previously associated with various social behavior alterations in animals. In cats, the inflammation of this organ, named vomeronasalitis, has been associated with the presence of aggression towards other cats. The aim of this study was to describe this pathology in farm pigs and to evaluate if it is associated with social alterations as described in cats. Our study showed that farm pigs can be affected by the inflammation of the vomeronasal organ and that the pigs with this alteration present an increased number of skin lesions induced by fights with other pigs. These results open an interesting perspective that links the alteration of this organ to the presence of aggressive behaviors in farm animals, which implicates an important issue in animal welfare.

**Abstract:**

The vomeronasal organ (VNO) is a bilateral chemosensory structure strongly involved in animal behaviour, thanks to its sensory epithelium (VNSE) that detects pheromones. Experimental VNO lesions can impair social, reproductive and maternal behaviour, while feline spontaneous vomeronasalitis has been associated with aggression. This study aimed to describe vomeronasalitis in farm pigs and explore its association with intraspecific behavioural alterations. Using 38 six-month-old pigs, the skin lesion score based on Welfare Quality^®^ protocols was obtained during the fattening period. The seventy-six VNOs from these pigs were stained in haematoxylin-eosin for histological examinations. VNSE inflammation was classified considering its intensity. Skin lesions data were compared to vomeronasalitis. There were 34% of pigs that showed unilateral VNSE inflammation, while 66% were bilaterally affected. The mean ± SD number of skin lesions/animal was 4.4 ± 2.82, and 34% of pigs scored 1 (moderately wounded animals) at least once during the fattening period. Statistical analysis showed an association between bilateral vomeronasalitis and skin lesion score (*p* < 0.05) and between bilateral moderate vomeronasalitis and skin lesions number (*p* < 0.01). This is the first report linking vomeronasalitis to social life in farm animals. Considering the role of social life in animal welfare, our data opens a research field linking pathology to animal behaviour.

## 1. Introduction

The vomeronasal organ (VNO) is a bilateral peripheral chemosensory structure that plays a pivotal role in the detection of pheromones in animals [1]. In pigs, the VNO key role in behaviour and chemical communication has been widely demonstrated by various studies [2,3,4]. As for the other species, porcine VNO is composed of a non-sensory epithelium (NSE) and a sensory epithelium (VNSE); only the VNSE contains receptor neurons that are able to detect pheromones [4]. In fact, from a histological and functional point of view, the VNSE strongly resembles the sensorial epithelium of the main olfactory system, while the NSE is comparable to the respiratory epithelium of the nasal cavity [5].

The critical role of the VNO in several aspects of animal behaviour has been widely demonstrated by studies focused on the experimental impairment of this organ. VNO surgical removal has been associated with deficits in reproductive behaviour in mice [6,7] and alarm pheromones detection in rats [8]. Moreover, the closure of the VNO nasoincisive duct has been linked to reproductive alteration in does [9] and deficits in maternal behaviour in sheep [10]. On the contrary, only a few pieces of evidence of spontaneous VNO alterations have been up to now described. In tree shrews, lymphocytic nodules were identified in VNO soft tissue, but the authors did not consider this change a pathological finding [11]. A similar observation has been recently reported in wallabies [12], while, in laboratory rabbits, the presence of VNO microabscesses was reported after the instillation of experimental solutions in the nasal cavity [13]. In mice, the VNO cells were shown as the Pseudorabies virus infection after experimental intranasal inoculation [14]. More recently, always in mice, the degenerative changes induced by the ageing process were characterized [15]. Finally, the relationship between vomeronasalitis and intraspecific aggressive disorders in domestic cats has been described [16], and it is the only report that links VNO spontaneous alterations to animal behavioural changes.

Intra-specific aggression in pigs could affect long-term social stress and have a negative impact on the welfare and productivity of pigs [17,18]. Aggressive behaviour is a component of the behaviour repertoire of both wild boar and pigs under commercial conditions. At mixing, aggressions occur for establishing a hierarchy between unfamiliar animals. Although aggression is most severe during the 24h post-mixing, a basal level of aggression persists thereafter in stable social groups, even when resource needs for survival are fully met [19].

The accumulation of skin lesions has been shown to reflect the involvement in aggressive behaviours, therefore skin lesions are commonly used to investigate the presence of aggression in farm pig groups [20]. The Welfare Quality^®^ (WQ) assessment protocol for pigs, which describes the procedures and requirements for the assessment of the welfare in farm pigs, also includes the indicator “wounds on the body” in the labelled animal welfare principle of “good health” [21].

As previously mentioned, the chemical messages play a critical role in vertebrates’ intraspecific communication, and the VNO is the structure responsible for their detection [1,22]. The aims of the present study were (1) to describe the VNO inflammation in pigs and (2) to assess if VNO inflammation was associated with skin lesions in stable groups of female pigs. It was hypothesized that VNO might be affected by inflammatory alterations, and these conditions may be associated with a higher number of skin lesions, which is indicative of chronic aggression in stable social groups.

## 2. Materials and Methods

### 2.1. Animals and Housing Conditions

Thirty-eight six-month-old female pigs ((Landrace × Large White) × Piétrain) from the experimental facilities of IRTA (Monells, Spain) were included in this study. Pigs were housed in slatted pens (5 × 2.7 m) provided with one steel drinker bowl (15 × 16 cm) connected to a nipple and a concrete feeder (58 × 34 cm) with four feeding places. Pigs had water and food ad libitum. Pigs were daily inspected, and no health problems were observed during the experimental period. Pigs were transported to the experimental slaughterhouse of IRTA (1.2 km trip) in pen groups, at 23 to 27 weeks of age (mean 108.0 ± SD 12.4 kg of body weight). Pigs were driven to a CO_2_ stunner and exposed to 90% for 3 min before exsanguination. Pigs snouts were obtained and fixed in 10% buffered formalin (pH 7.4) for histopathological analysis. The study was reviewed and approved by the Institutional Animal Care and Use Committee of IRTA and Generalitat de Catalunya (protocol number 7622).

### 2.2. Skin Lesions

Skin lesions were assessed based on the Welfare Quality^®^ assessment protocol (http://www.welfarequalitynetwork.net/media/1018/pig_protocol.pdf, accessed on 30 November 2021) for pigs [18], 3 times during the fattening period (at 4, 5 and 6 months of age). One side of the pigs’ body was inspected visually for the presence of fresh scratches (fresh blood, bright red and longer than 2 cm), considering 5 separate zones: ear, front, middle, hind-quarters and legs. The number of skin lesions per zone and pig was obtained. Additionally, pigs were scored as 0 (no wounded animals) if all regions of its body had a maximum of 4 scratches. Pigs were scored as 1 (moderately wounded animals) when from 5 to 10 scratches were observed at least in 1 zone or a maximum of 1 body region with 11 to 15 scratches. Pigs were scored as 2 (severely wounded animals) when more than 10 scratches were observed on a minimum of 2 zones of the body or if any zone had more than 15 scratches.

### 2.3. Histopathological Analysis

The 76 VNOs from the 38 pigs were extracted from the 38 snouts, processed by routine methods and paraffin-embedded for the histological analysis. VNOs were cut transversally to respect their length in order to have serial sections and exhaustively observe both the NSE and the VNSE at the histological exam. Four-µm-thick sections were cut and stained with hematoxylin and eosin for the histopathological investigation. VNSE and NSE inflammations were classified depending on the intensity of the process by weak (score 1), moderate (score 2) and strong (score 3) inflammation, and by acute or chronic, according to the type of cellular infiltrate [16]. Due to the different roles played by NSE and VNSE [4], only VNSE changes were included in statistical tests. Laterality of VNSE inflammation was also considered. According to the histological exam, three pathological conditions were assessed: (i) absence/presence of bilateral VNSE inflammation, independently from alteration intensity; (ii) absence/presence of bilateral moderate VNSE inflammation; and (iii) right plus left scores total. Pigs were scored as follows: 1 = 1 weakly inflamed VNSE; 2 = 2 weakly inflamed or 1 moderately inflamed VNSEs; 3 = 1 weakly and 1 moderately inflamed VNSE; 4 = 2 moderately inflamed VNSE.

### 2.4. Statistical Analysis

The VNSE pathological results were compared with the mean value of three skin lesions measurements (as quantitative data) and skin lesion score (as qualitative data). Statistical analysis was performed using 9.4 SAS software (SAS Institute Inc., Cary, NC, USA). As the mean number of skin lesions presented neither a normal distribution nor homoscedasticity, the Wilcoxon two-sample test and the Kruskal–Wallis one way ANOVA were used using the npar1way procedure. The skin lesion score was compared to pathological variables using the Fisher exact test due to the small size of the samples using the freq procedure. Statistical significance was based on a 5% (0.05) significance level.

## 3. Results

### 3.1. Skin Lesions

The mean number of the 3 skin lesions measurements was 4.4 (SD: 2.82; range: 1–11.7). Regarding the skin lesion score, 25/38 subjects (65.8%) scored 0 in all the assessments, while 13/38 (34.2%) scored 1 at least once during the studied period. No animals scored 2 in any measurement. Data concerning skin lesions and skin lesion scores are reported in Table 1, individual data are reported in supplementary material Table S1.

### 3.2. Histopathological Analysis

**Figure 1:**
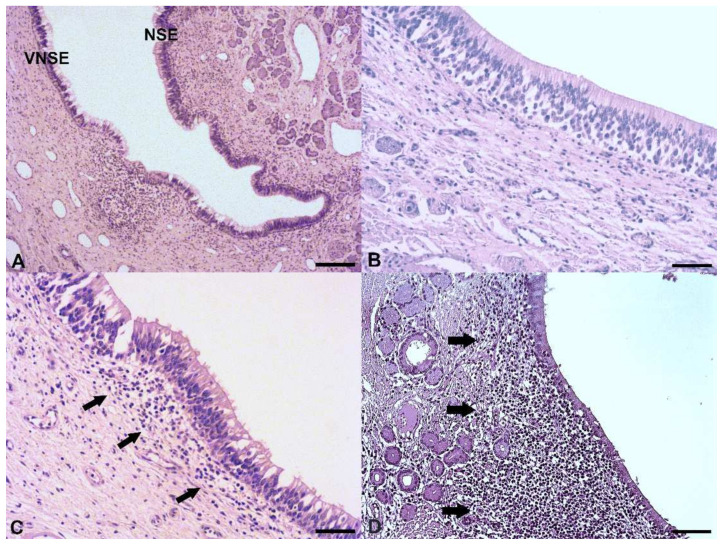
Pig vomeronasal organ. (**A**) Porcine chronic vomeronasalitis affecting the vomeronasal sensory epithelium (VNSE) and non-sensory epithelium (NSE). Hematoxylin and eosin stain. Scale bar, 150 µm. (**B**) Healthy VNSE. Hematoxylin and eosin stain. Scale bar, 50 µm. (**C**) Weak chronic vomeronasalitis affecting porcine VNSE. A weak inflammatory infiltrate (arrows) is observable under the epithelium. Hematoxylin and eosin stain. Scale bar, 50 µm. (**D**) Moderate chronic vomeronasalitis affecting porcine VNSE. A dense inflammatory infiltrate, mainly composed of small and mature lymphocytes (arrows), is observable under the epithelium and among the vomeronasal glands. Hematoxylin and eosin stain. Scale bar, 100 µm.

Of the 76 VNOs included in the study, the VNSE did not present pathological changes in 13 samples (17.1%), while in 31/76 (40.8%) and 32/76 (42.1%), the VNSE was affected by chronic inflammation of weak and moderate intensity, respectively. The NSE was always affected by chronic inflammation, of weak intensity in 30/76 (39.5%) and moderate-intensity in 46/76 (60.5%) pigs. No VNOs presented inflammation of strong entity in the VNSE or NSE portion. Inflammatory infiltrate was mainly located in the soft tissue under epithelia and was mostly composed of mature small lymphocytes (Figure 1). Plasma cells and macrophages were present in smaller quantities, while mast cells and mature non-degenerate neutrophils were only rarely observable. Moreover, neutrophils were almost exclusively located among epithelial layers. In moderate inflammations, inflammatory cells arrived to infiltrate vomeronasal nerves and glands. The different intensities of VNSE inflammation are illustrated in Figure 1.

There were 13/38 (34.2%) subjects who presented unilateral VNSE inflammation, while in 25/38 (65.8%) VNSE inflammation was bilateral (supplementary material: Table S1). Of the bilaterally affected, 12/38 (31.6%) pigs presented a moderate bilateral VNSE inflammation. The distribution and intensity of VNSE inflammation is reported in Table 2.

Summing up the VNSE inflammatory score from each VNO for each pig, we obtained the following groups: 11/38 (28.9%) pigs scored 1 (group 1), 9/38 (23.7%) scored 2 (group 2), 6/38 (15.8%) scored 3 (group 3) and 12/38 (31.6%) scored 4 (group 4).

### 3.3. Association between Skin Lesions and Vomeronasalitis

**Figure 2:**
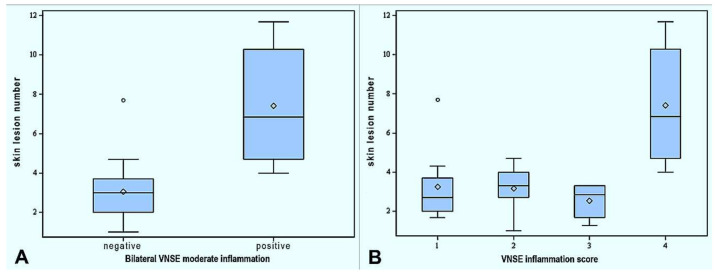
(**A**) Box plot showing the mean number of skin lesions in pigs bearing bilateral VNSE moderate inflammation and pigs bearing weaker alterations. Pigs with bilateral VNSE moderate inflammation (“positive” in the figure) presented a significant increase in skin lesions (*p* = 0.0001). (**B**) Box plot showing the mean number of skin lesions in the four groups obtained considering the VNSE inflammation score. Pigs scored as 4 presented significantly more skin lesions than the other 3 groups (*p* = 0.0001). No differences were observed between pigs scored as 1, 2 and 3 (*p* > 0.05).

Regarding the mean number of skin lesions, there was no difference between pigs with unilateral VNSE inflammation and those bilaterally affected (*p* = 0.10), if the intensity of the process was not considered. On the contrary, an association between the presence of bilateral moderate VNSE inflammation and the mean number of skin lesions (*p* = 0.0001) was observed. Pigs affected by bilateral moderate VNSE inflammation presented a mean skin lesions number of 6.85, while the other subjects were 3.0, as shown in Figure 2A. Considering the total VNSE inflammation score, pigs scored as 4 presented more skin lesions than all the other groups (pigs scored as 1: 2.7, pigs scored as 2: 3.3, pigs scored as 3: 2.85, pigs scored as 4: 6.85; *p* = 0.0001;), as reported in Figure 2B. No differences were observed between pigs scored as 1, 2 and 3 (*p* > 0.05).

An association between skin lesion score 1 and all the histopathological parameters was detected: bilateral VNSE inflammation (*p* < 0.05), bilateral moderate VNSE inflammation (*p* < 0.01) and VNSE total score (*p* < 0.01). In particular, the pigs scored as 4 for VNSE inflammation correlated with skin lesion score 1 if compared with pigs scored as 1 (*p* = 0.0004), pigs scored as 2 (*p* = 0.0019) and pigs scored as 3 (*p* = 0.0128). No differences were observed comparing scored as 1, 2 and 3 for VNSE inflammation between them (*p* > 0.05).

## 4. Discussion

This report described for the first time spontaneous VNO inflammation in farm animals, specifically in pigs. Moreover, bilateral VNO inflammation was associated with skin lesions, which are indicative of aggression. Up to now, this phenomenon has been described only in cats by previous research in which we found an association between VNSE inflammation and intraspecific aggression [16].

As in cats, we observed that the porcine VNO was affected only by chronic inflammations, with no cases of acute and suppurative [16]. Intensive farm pigs are chronically exposed to several environmental contaminants, such as dust, ammonia and hydrogen sulfide, which can induce respiratory tract diseases [23,24]. The respiratory system is constantly exposed to these contaminants, and it is suggested that the VNO can also be the target, as its anatomical position and histomorphology is partially comparable to the nasal cavity [5]. Furthermore, from a macroscopic and histological point of view, all the lesions that we observed were not attributable to a specific pathogen. On the other hand, the absence of acute vomeronasalitis in our population may be due to the fact that all the animals/pigs were slaughtered at the same age, thus after six months of exposition to the previously mentioned contaminants, when the acute phase was already passed, and the inflammation switched to chronic. In this study, the presence of the VNO alteration was assessed by post-mortem histology. Further studies should also investigate the possibility to detect this kind of alteration in living animals to detect earlier and eventually treat them (with non-steroidal anti-inflammatory drugs, for example). The current bibliography on this topic is inexistent, except for a recent study in which the authors could clearly visualize the VNO by magnetic resonance imaging in dogs [25]. This technique seems to be a promising tool to assess VNO conditions in vitam.

The importance of chemical communication in vertebrates’ life has been widely recognized, as well as the role of the VNO in detecting this kind of message [1,22]. Intraspecific communication plays a role even more critical in farm animals due to the increased density condition in intensive pig production systems [26]. In fact, these conditions lead to frustration-induced behaviors, with the consequent increase of intraspecific aggression and problems in the environmental adaptation process. The pigs assessed in the present study were allocated in experimental conditions (with densities from 0.7 m^2^/pig to 1 m^2^/pig). Consequently, it is expected that inflammation of the VNO could be even more intense in pigs allocated in commercial conditions (with densities of 0.65 m^2^/pig until 110 kg body weight).

Even if further studies should be carried out to better verify the possible cause-effect relation, the statistical analysis revealed that skin lesions were associated with the presence of bilateral inflammation of the VNSE, the VNO portion responsible for chemical messages detection [5]. More precisely, moderate VNSE changes reduced welfare conditions more than weak inflammation, as a probable consequence of the increasing impairment action carried out by the inflammatory microenvironment. In humans, olfactory dysfunction has been associated with olfactory mucosa inflammation [27], and some cytokines have been recognized as directly involved in olfactory loss during human and mouse chronic inflammation, such as the tumor necrosis factor-alpha (TNF-α) and the interleukin 6 (IL-6) [28,29,30]. Considering the morphological and functional similarities between the olfactory mucosa and the VNSE [5], it is the authors’ opinion that such microenvironment could also be at the base of VNO dysfunction during porcine vomeronasalitis, as we previously suggested in cats [16]. In particular, TNF-α impairs mouse olfaction through reduction of neurons number, olfactory epithelium thickness and nervous function [28,29].

Some interesting data is that unilateral VNSE inflammation does not seem to influence pig behavior. In fact, only the bilateral VNSE inflammation was associated with increased skin lesions. It is suggested that one healthy VNO can guarantee a normal semiochemicals detection in this species, supplying to the dysfunction of the affected VNO. On the contrary, pigs with bilateral VNSE alterations seem to present reduced chemical communication capabilities, a condition that could increase the risk of social disorganization, making these animals more exposed to other pigs’ aggression.

Furthermore, it is well established that animal behavior and animal welfare are two very strictly linked aspects of animals’ life [31], and the health status of a population can impact the level of welfare. For these reasons, it is the authors’ opinion that this first description of porcine vomeronasalitis could also be considered as a welfare problem. As mentioned before, high animal density is a critical factor in animal welfare worsening, as this condition facilitates contact and aggressive behaviors among pigs [26]. Moreover, animal density plays a key role also in increasing farm contaminants concentration, such as ammonia, hydrogen sulfide and dust, which are commonly responsible for respiratory inflammation [23,24] and potentially for vomeronasalitis. Thus, during a complex situation in which pigs must constantly communicate among them, they could not fully dispose of one of the most important types of intraspecific communication, as the chemical messages are [1,22]. Due to the multifactorial nature at the base of animal welfare and behavior, one of the most important implications emerging from our study is that the vomeronasalitis could contribute to animal welfare worsening, as a cofactor of the other causes reported until now in the literature [26]. Even if our results should be confirmed by further studies focused on farm animal vomeronasalitis and its implications, this study may open an innovative way of research in support of animal welfare.

## 5. Conclusions

To the best of our knowledge, here we described for the first time the evidence of vomeronasalitis in farms pigs and its potential role in influencing animal behavior and welfare. Even if other studies are needed before drawing firmer conclusions, these results could reveal a number of critical implications, considering the pivotal role of this organ in several aspects of animal life. Furthermore, the description of chronic vomeronasalitis in pigs highlights the importance to consider healthy environmental farm conditions in order to prevent animal welfare problems.

## Figures and Tables

**Table 1:** Descriptive statistics of mean number of skin lesions according to zone of the body assessed (ear, front, middle, back, legs) and all body regions in the 38 pigs studied.

Body Zone	Mean	SD
Ear	0.3	0.36
Front	1.7	1.31
Middle	1.2	1.23
Back	1.1	1.12
Legs	0.1	0.22
All body regions	4.4	2.80

**Table 2:** Laterality and intensity of VNSE inflammation in the 38 pigs included in this study.

Laterality and Intensity of VNSE Inflammation. (First VNO + Second VNO)	Number of Cases	Percent (%)
Healthy	0/38	0
Unilateral VNSE chronic inflammation	13/38	34.2
healthy (score 0) + weak inflammation (score 1)	11/13	84.6
healthy (score 0) + moderate inflammation (score 2)	2/13	15.4
Bilateral VNSE chronic inflammation	25/38	65.8
weak (score 1) + weak inflammation (score 1)	7/25	28.0
weak (score 1) + moderate inflammation (score 2)	6/25	24.0
moderate (score 2) + moderate inflammation (score 2)	12/25	48.0

## Data Availability

The data presented in this study are available within the article in the supplementary Materials.

## Supplementary Materials

The following supporting information can be downloaded at: https://www.mdpi.com/article/10.3390/ani12030303/s1, Table S1: individual data of skin lesion and VNO inflammation parameters. The data presented in this study are available within the article in the supplementary files.
